# Selective Myeloid Depletion of Galectin-3 Offers Protection Against Acute and Chronic Lung Injury

**DOI:** 10.3389/fphar.2021.715986

**Published:** 2021-08-30

**Authors:** Duncan C. Humphries, Ross Mills, Ross Dobie, Neil C. Henderson, Tariq Sethi, Alison C. Mackinnon

**Affiliations:** ^1^ Centre for Inflammation Research, University of Edinburgh, Edinburgh, United Kingdom; ^2^ Galecto Inc, Copenhagen, Denmark

**Keywords:** galectin-3, neutrophil, myeloid cells, depletion, lung, injury

## Abstract

**Rationale:** Galectin-3 (Gal-3) is an immune regulator and an important driver of fibrosis in chronic lung injury, however, its role in acute lung injury (ALI) remains unknown. Previous work has shown that global deletion of galectin-3 reduces collagen deposition in a bleomycin-induced pulmonary fibrosis model (MacKinnon et al., Am. J. Respir. Crit. Care Med., 2012, 185, 537–46). An inhaled Gal-3 inhibitor, GB0139, is undergoing Phase II clinical development for idiopathic pulmonary fibrosis (IPF). This work aims to elucidate the role of Gal-3 in the myeloid and mesenchymal compartment on the development of acute and chronic lung injury.

**Methods:***LgalS3*^fl/fl^ mice were generated and crossed with mice expressing the myeloid (*LysM*) and mesenchymal (*Pdgfrb*) cre drivers to yield *LysM-cre* ^*+/-*^ */LgalS3* ^*fl/fl*^ and *Pdgfrb-cre* ^*+/-*^ */LgalS3* ^*fl/fl*^ mice. The response to acute (bleomycin or LPS) or chronic (bleomycin) lung injury was compared to globally deficient *Gal-3* ^*−/−*^ mice.

**Results:** Myeloid depletion of Gal-3 led to a significant reduction in Gal-3 expression in alveolar macrophages and neutrophils and a reduction in neutrophil recruitment into the interstitium but not into the alveolar space. The reduction in interstitial neutrophils corelated with decreased levels of pulmonary inflammation following acute bleomycin and LPS administration. In addition, myeloid deletion decreased Gal-3 levels in bronchoalveolar lavage (BAL) and reduced lung fibrosis induced by chronic bleomycin. In contrast, no differences in BAL Gal-3 levels or fibrosis were observed in *Pdgfrb-cre* ^*+/-*^ */LgalS3* ^*fl/fl*^ *mice.*

**Conclusions:** Myeloid cell derived Galectin-3 drives acute and chronic lung inflammation and supports direct targeting of galectin-3 as an attractive new therapy for lung inflammation.

## Introduction

Galectin-3 (Gal-3) is a pro-fibrotic, mammalian ß-galactoside binding lectin found to be highly upregulated in the injured lung, particularly in patients with idiopathic pulmonary fibrosis (IPF) suffering acute exacerbations (MacKinnon et al., 2012). Previous work has shown that global deletion of Gal-3 reduces collagen deposition in a bleomycin-induced pulmonary fibrosis model (MacKinnon et al., 2012). Gal-3 has been shown to stimulate migration and collagen synthesis in fibroblasts (Nishi et al., 2007) whilst regulating alternative, pro-fibrotic, macrophage activation (Mackinnon et al., 2008). GB0139 (formerly TD139), a novel, inhaled, small molecule Gal-3 inhibitor developed by Galecto Biotech, reduces fibrosis severity following bleomycin administration (MacKinnon et al., 2012; Delaine et al., 2016), and targets lung macrophages in IPF (Hirani et al., 2021). GB0139 is currently undergoing a Phase IIb clinical trial for the treatment of IPF (NCT03832946).

Despite its known contribution during chronic lung disease, the role of Gal-3 in the development of acute lung injury (ALI) remains unclear. In humans, Gal-3 is a marker of airway inflammation in bronchial asthma (Elkolaly and Ali, 2018) and is elevated in patients with chronic obstructive pulmonary disease (COPD), where it is associated with neutrophil accumulation in the small airways (Pilette et al., 2007). It has also recently been implicated for its role in inflammatory and fibrotic responses following COVID-19 infection (Garcia-Revilla et al., 2020).

Gal-3 also acts as a pro-inflammatory mediator in acute colitis (Simovic Markovic et al., 2016) and neuroinflammation (Burguillos et al., 2015) where it promotes NOD-like receptor family, pyrin domain containing 3 (NLRP3) inflammasome activation and IL-1β production in macrophages (Simovic Markovic et al., 2016), whilst acting as a ligand for Toll-like receptor 4 (TLR4) (Burguillos et al., 2015). In alveolar epithelial cells (AECs), Gal-3 activates ERK, AKT and JAK/STAT1 signalling pathways, leading to dysregulated pro-inflammatory cytokine release during influenza and *Streptococcus pneumoniae* co-infection (Nita-Lazar et al., 2015) and enhances the pathogenic effects of H5N1 avian influenza virus by promoting excessive host inflammatory responses via macrophage NLRP3 inflammasome activation (Chen et al., 2018). Mice deficient in Gal-3 develop less severe inflammation and IL-1β production than wild type (WT) mice (Chen et al., 2018).

In this study, we aimed to elucidate the major cell types involved in Gal-3 mediated acute (mild and moderate) and chronic lung inflammation. We used the LysM and Pdgfrb promoters (Foo et al., 2006) to generate mice with a specific deletion in the myeloid or mesenchymal compartment, respectively. The *LysM-cre* targets predominately lung macrophages but also neutrophils and dendritic cells (McCubbrey et al., 2017) while *Pdgfrb-Cre* has been shown to effectively target recombination in PDGFRß^+^ mesenchymal cells in liver, lung and skeletal and cardiac muscle (Henderson et al., 2013). Mice with a specific depletion of Gal-3 in the myeloid (*LysM-cre* ^*+*^ */Lgals3* ^*fl/fl*^) or the mesenchymal (*Pdgfrb-cre* ^*+*^ */Lgals3* ^*fl/fl*^) compartment were generated and compared with responses in Gal-3 expressing and globally deficient mice with LPS/bleomycin injury. We show that selective myeloid depletion decreased lung Gal-3 levels and diminished acute lung injury *via* a reduction in neutrophil recruitment and inflammation as well as reducing bleomycin-induced fibrosis, whereas depletion in the mesenchymal compartment had no effect. Some of the results of these studies have been previously reported in the form of an abstract (Mackinnon et al., 2019).

## Methods

### Generation of Genetically Modified Animals

All animal experimental work was carried out under a project license approved by the local Animal Welfare and Ethical Review body (AWERB) and issued in accordance with the Animals (Scientific Procedures) Act 1986. C57BL/6 were purchased from Harlan Laboratories. Generation of *Gal-3* ^*−/−*^ mice by gene-targeting technology has been described previously (Hsu et al., 2000). LgalS3^flox/flox^ mice (C57BL/6N-Lgals3^tm1c(EUCOMM)Wtsi^/H mice were generated as described (Maupin et al., 2018) from an ES cell clone (clone I.D. EPD0377_1_A09) by MRC Harwell UK). Transgenic mice with selective depletion of Gal-3 in myeloid or mesenchymal cells were created by cross breeding *LysM-cre* mice (kindly provided by Dr. Luca Cassetta Edinburgh UK) or *Pdgfrb-cre* mice (provided by Ralf Adams, University of Münster, Germany) (Foo et al., 2006) with LgalS3^flox/flox^ mice, respectively, to yield *LysM-cre* ^*+/-*^ */LgalS3* ^*fl/fl*^ mice and *Pdgfrb-cre* ^*+/-*^ */LgalS3* ^*fl/fl*^ *mice*.

### Induction of lung Inflammation

10 µg Lipopolysaccharide (LPS, serotype 0127:B8, L4516, Sigma-Aldrich, Missouri, United States) or 33 µg bleomycin (BI3543, Apollo Scientific, UK) in 50 µl 0.9% NaCl, was instilled *intra-tracheally (i.t.)*. Lungs were examined at 24 and 48 h (bleomycin/LPS) or 21 days post bleomycin. Blood was obtained via the vena cava. Bronchoalveolar lavage *(*BAL) was collected (3 ml x 0.8 ml PBS) to retrieve cells from the alveolar compartment. Lungs were then perfused with 2 ml sterile saline via the right ventricle prior to removal.

### Histology and Immunohistochemistry

Lungs were fixed in formalin and embedded in paraffin-wax prior to sectioning and staining with haematoxylin and eosin or Masson’s trichrome. Inflammation and fibrosis was determined using published methods (Murao et al., 2003; Hübner et al., 2008). For immunohistochemistry, sections underwent antigen retrieval using 10 mM sodium citrate buffer pH 6.0. The following primary antibodies were used; PDGFRβ (rabbit anti human/mouse clone Y92, 1:500, ab32570, Abcam), Gal-3 (FITC-conjugated anti-Gal-3 CL8942F, Cedarlane). For Gal-3 sections were incubated with an anti-rat biotin-conjugated secondary (BA-4001-5, Vectorlabs) and visualised with DAB chromogen using a DS9800 Leica Biosystem. Slides were scanned using an Axioscan microscope with a 20x lens. For immunofluorescent staining slides were incubated sequentially with anti-PDGFRβ followed by donkey anti-rabbit HRP polymer and amplified with tyramide OPAL 650 (Okoyabio). Slides underwent antigen retrieval prior to incubation with anti-Gal-3 and goat anti-rat Alexa Fluor 488 (Molecular Probes). Slides were mounted in fluorescent mounting medium (Dako) and imaged on a Zeiss Axio imager z1 fluorescence microscope.

### BAL Analysis

Total protein within BAL was performed using a Pierce BCA Total Protein Assay Kit (23227; ThermoFisher Scientific). Gal-3 was measured by enzyme-linked immunosorbent assay [ELISA (mouse Gal-3 DuoSet; R&D Systems)] as per the manufacturers’ instructions. Cytokine profiles were assessed using the mouse magnetic luminex assay (LXSAMSM, R&D Systems, Minneapolis, MN, United States) as per the manufacturers’ instructions.

### Flow Cytometric Analysis of Lung Digests, BAL and Blood Cells

Cellular analysis of lung digests was performed according to published methodology (Humphries et al., 2018). In brief, minced lungs were digested with 0.1 mg/ml DNase 1 (DN-25, Sigma-Aldrich) and 1 mg/ml Collagenase D (11088866001, Roche) at 37°C for 1 h. Cells were resuspended in PBS and strained through a 40 μm cell strainer (352340, BD Biosciences). Red cells were lysed with cold ACK buffer (Ammonium-Chloride-Potassium, A10492-01, Invitrogen). Lung digest, BAL and blood cells were blocked with Fc block™ (1:100) for 10 min at 4°C prior to antibody staining for 30 min on ice. Cells were fixed with FACS lysing solution (349202, BD Biosciences) prior to flow cytometric analysis using an LSR Fortessa (BD Biosciences). Data analysis was performed using FlowJo software, version 7.2.4 (Tree Star Inc., United States).

The following antibodies were used from BD Biosciences or Biolegend: BV605 anti-mouse CD11b, eFluoro-450 anti-mouse CD11b, PE-Cy7 anti-mouse CD11c, AF700 anti-mouse CD11c, APC anti-mouse CD24, BV650 anti-mouse CD45, PE anti-mouse CD64, BV421 anti-mouse CD64, APC-Cy7 anti-mouse Ly-6C, PerCP anti-mouse Ly-6C, Alexa-Fluor 700 anti-mouse Ly-6G, BV711 anti-mouse Ly-6G, PerCP-Cy5.5 anti-mouse MHC II, PE anti-mouse CD80, PE-Cy7 anti-mouse CD206, PE anti-mouse Siglec-F, PE-Cy7 anti-mouse CD3, PE anti-mouse CD4, Alexa Fluor 700 anti-mouse CD8, Pacific Blue anti-mouse B220, Live/Dead fixable aqua, and FITC anti-mouse Gal-3 (Cedarlane Labs).

### Bone Marrow Cell Isolation

Bone marrow was flushed from the femurs of *LysM-cre* ^*+/-*^ mice using DMEM (ThermoFisher Scientific, Massachusetts, United States) and processed for flow cytometric analysis.

### Total Lung collagen

Left lobe total collagen levels were quantified using the soluble (S1000, Biocolor, United Kingdom) and insoluble (S2000, Biocolor) sircol assays as per the manufacturers’ instructions.

### Statistics

Data are represented as mean ± the standard error of the mean (SEM). Quantification of histology/immunohistochemistry was performed blinded by the investigator. Statistical comparisons were made using two-tailed Students t-test or one-way/two-way analysis of variance (ANOVA) with Bonferroni post-test for multiple comparisons. A *p*-value of less than 0.05 was considered statistically significant (* = *p* < 0.05, ** = *p* < 0.01, *** = *p* < 0.001). All graphs and statistics were performed using the statistical package GraphPad Prism 5 for Windows (GraphPad Software, California, United States).

## Results

### Generation of Mice With Myeloid and Mesenchymal Conditional Gal-3 Deletion

**FIGURE 1:**
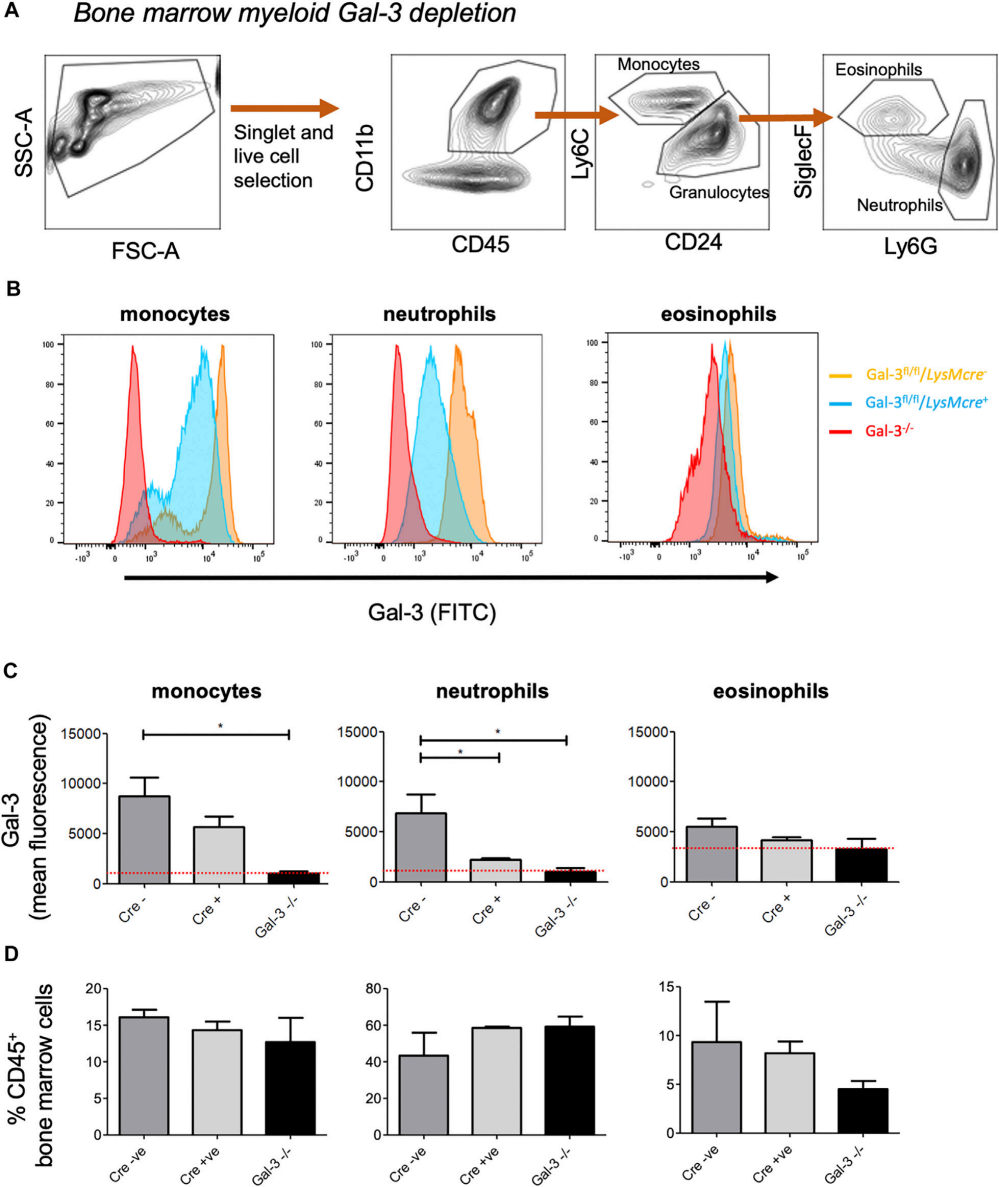
*–*Confirmation of Myeloid cell Gal-3 depletion using bone marrow-derived leukocytes. **(A)** Gating strategy to identify monocytes (CD45^+^, CD11b^+^, Ly6C^+^, CD24^−^), neutrophils (CD45^+^, CD11b^+^, Ly6C^−^, CD24^+^, Ly6G+, SiglecF^−^) and eosinophils (CD45^+^, CD11b^+^, Ly6C^−^, CD24^+^, Ly6G^−^, SiglecF^+^) in bone marrow leukocytes. **(B)** Gal-3 expression in bone marrow-derived leukocytes. Gal-3 expression on was assessed via flow cytometry. **(C)** Quantification of Gal-3 expression on monocytes, neutrophils and eosinophils. Red line represents mean fluorescence intensity (MFI) in Gal-3^−/−^ mice. **(D)** Proportion of monocytes, neutrophils and eosinophils in the bone marrow. Data represented as mean ± SEM. Analysed via 1-way ANOVA (*n* = 6, *p < 0.05, **p < 0.01, ***p < 0.001).

Lgals3 flox mice (Lgals3^fl/fl^) were generated as described by Maupin et al. (2018) and crossed with mice expressing either the *LysM-cre* or the *Pdgfrb-cre* transgene to obtain mice with a new genomic Lgals3-null allele in the myeloid or mesenchymal compartment. Gal-3 depletion was initially examined in bone-marrow derived cells from *LysM-cre* ^*+*^ *, LysM-cre* ^*-*^ and *Gal-3* ^*-/-*^ mice (Figures 1A–C). Monocytes and neutrophils obtained from the bone marrow of *LysM-cre* ^*+*^ mice exhibited a 35 and 75% reduction in surface Gal-3 expression, respectively, when compared to *LysM-cre* ^*-*^ mice, indicating predominate depletion in neutrophils following recombination. No changes were observed in eosinophils, which displayed low levels of Gal-3. The apparent higher mean fluorescence in Gal-3^−/−^ eosinophils is due to increased cellular autofluorescence in those cells. There was no difference in the frequency of monocytes, neutrophils or eosinophils between the genotypes (Figure 1D).

### Bleomycin-Induced Acute Lung Injury

**FIGURE 2:**
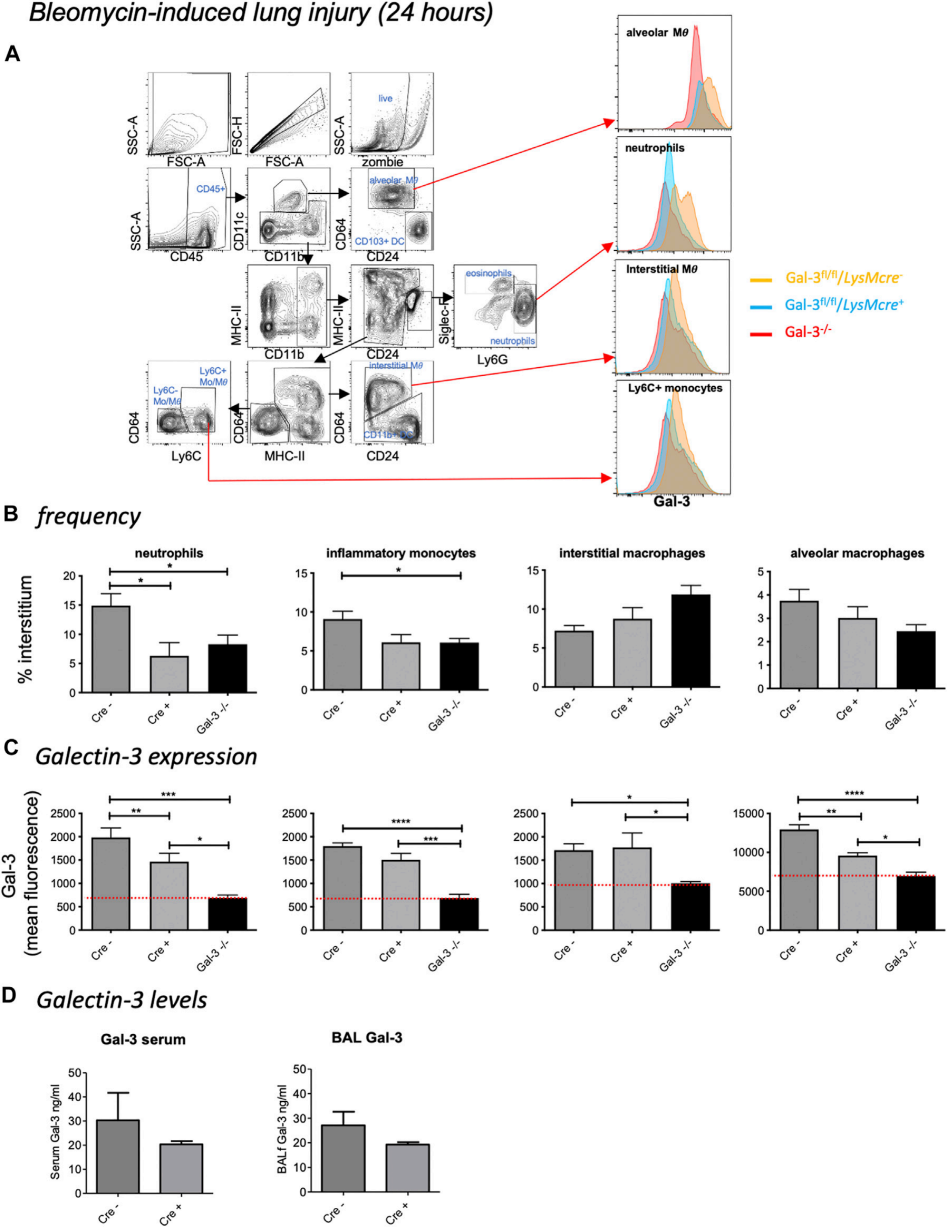
Bleomycin-induced acute lung injury. Mice received 33 µg bleomycin and were retrieved at 24 h. **(A)** Flow cytometric gating strategy to identify immune cell subsets and Gal-3 expression within the lung. After gating for single, live cells, leukocytes were selected based on CD45 expression. Alveolar macrophages (MΦ) (Siglec F^+^ CD11b^int^ CD11c^+^ CD64^+^), CD103^+^ dendritic cells (DCs - CD11c^+^ CD103^+^ CD24^+^), neutrophils (CD11b^+^ Ly-6G^+^) and eosinophils (SiglecF^+^ CD11b^+^ CD11c^–^), followed by identification of the populations with overlapping expression patterns: interstitial macrophages (CD11b^+^ MHC II^+^ CD11c^+^ CD64^+^ CD24^–^), CD11b^+^ DCs (CD11b^+^ MHC II^+^ CD11c^+^ CD24^+^ CD64^–^), inflammatory monocytes (CD11b^+^ MHC II^−^CD64^−^ Ly-6C^hi^) and patrolling monocytes (CD11b^+^ MHC II^−^CD64^−^ Ly-6C^low^). **(B)** Interstitial leukocyte frequencies following acute bleomycin. **(C)** Galectin-3 expression in leukocyte subsets. Red line represents mean fluorescence intensity (MFI) in *Gal-3* ^*−/−*^ mice. d) Gal-3 levels in serum and BAL. Data represented as mean ± SEM. Analysed via 1-way ANOVA (b,c) or students *t*-test. **(D)** (*n* = 6, *p < 0.05, **p < 0.01, ***p < 0.001).

To confirm Gal-3 knockdown in various pulmonary leukocyte subsets, mice received 33 µg bleomycin and were retrieved at 24 h (for gating strategy see Figure 2A). Despite inducing mild inflammation, a reduction in neutrophil recruitment was observed in *LysM-cre* ^*+*^ and *Gal-3* ^*−/−*^ mice compared to *LysM-cre* ^*-*^ mice following acute bleomycin administration (Figure 2B). Compared to globally deficient *Gal-3* ^*−/−*^ mouse lung digests, Gal-3 expression was only significantly reduced by 26% in both neutrophils and alveolar macrophages in mice bearing the conditional deletion, with overall expression found to be highest on alveolar macrophages (Figure 2C). Surprisingly, inflammatory (Ly-6C^hi^) monocytes, patrolling (Ly-6C^low^) monocytes (Supplementary Figures S1A,B) and interstitial macrophages did not exhibit significantly reduced Gal-3 expression in the *LysM-cre* ^*+*^ mice. Gal-3 levels for all cell types observed were significantly lower in *Gal-3* ^*−/−*^ mice compared to *LysM-cre* ^*+*^ *,* indicating incomplete depletion in *LysM-cre* ^*+*^ neutrophils and alveolar macrophages. No changes in eosinophil, CD103^+^ dendritic cell (DC) or CD11b^+^ DC Gal-3 expression were observed in *LysM-cre* ^*+*^ mice (Supplementary Figures S1A,B). B cells, CD4^+^ T cells and CD8^+^ T cells exhibited low Gal-3 expression in all genotypes and were not affected by the presence of the transgene (Supplementary Figure S1C). However there was a 35% reduction in Gal-3 expression in the CD45^−^ cell population which would comprise primarily lung epithelial cells in *LysM-cre* ^*+*^ mice (Supplementary Figure S1D), suggesting that the LysM promotor also partially targets the epithelium which is in keeping with other studies (McCubbrey et al., 2017). Alveolar macrophages and the epithelium are the major sources of Gal-3 in the lung.

Galectin-3 was detectable in both serum and bronchoalveolar lavage fluid (BALf) of *LysM-cre* transgene mice (Figure 2D). No significant differences in Gal-3 levels were observed between the conditional knockout mice, however similar trends towards reduced expression was seen in both serum and BAL in *LysM-cre* ^+^ mice. A trend of reduced pro-inflammatory cytokine levels (IL-6 and G-CSF) were also observed in *Gal-3* ^*−/−*^ mice with a significant reduction in G-CSF in *LysM-cre* ^*+*^ mice compared to *LysM-cre* ^*-*^ (Supplementary Figure S2). No differences were observed in the anti-inflammatory cytokine IL-10.

### LPS-Induced Acute Lung Injury

**FIGURE 3:**
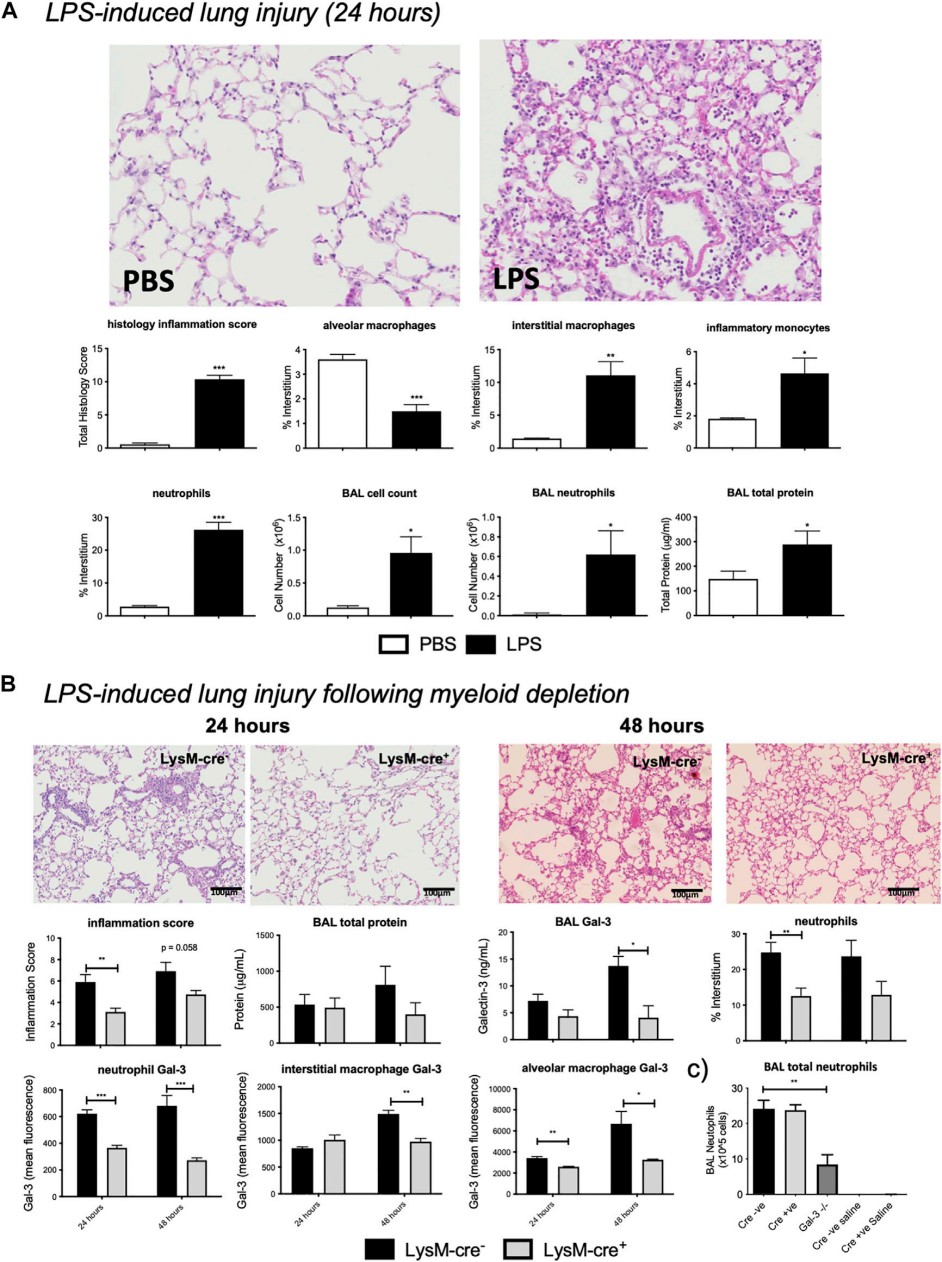
*–* LPS induced acute lung injury. **(A)** LPS injury assessment in *LysM-cre* ^*-*^ mice. *LysM-cre* ^*-*^ mice received either PBS or 10 µg LPS and were retrieved 24 h later. **(B)** *Effect of myeloid cell Gal-3 depletion on LPS-induced lung inflammation.* 10µg LPS was administered *i. t.* to *LysM-cre* ^*-*^ and *LysM-cre* ^*+*^ mice and retrieved at 24 or 48 h. Injury levels were analysed via histological inflammation score, tissue digest and BAL assessment. **(C)** BAL total neutrophils in *LysM-cre* ^*-*^, *LysM-cre* ^*+*^ and *Gal-3* ^*−/−*^ mice following 24 h LPS or saline control. Data represented as mean ± SEM. Analysed via students t-test (*n* = 4–5, **p < 0.01, ***p < 0.001). Images taken at x200.

Moderate pulmonary inflammation was then assessed using the LPS model of ALI. LPS administration resulted in significant pulmonary inflammation at 24 h, as seen by alveolar membrane thickening, capillary congestion, intra-alveolar haemorrhage, and interstitial and alveolar neutrophil infiltration (Figure 3A). Flow cytometric analysis of lung digests revealed an increase in interstitial macrophage, inflammatory monocyte and neutrophil recruitment alongside a reduction in alveolar macrophages following LPS administration. BAL cell counts were elevated resulting from alveolar neutrophil infiltration which corresponded with increased total protein levels.

### Myeloid Cell-Derived Gal-3 Partially Reduces LPS-Induced Acute Lung Injury

To assess the contribution of myeloid cell-derived Gal-3, the LPS model of ALI was assessed in *LysM-cre* mice (Figure 3B). Histology inflammation score was significantly reduced in *LysM-cre* ^*+*^ mice 24 h after LPS administration, with a further reduction seen at 48 h that correlated with a reduction in interstitial neutrophil accumulation. BALf Gal-3 increased with time in *LysM-cre* ^*-*^ mice but was significantly reduced at 48 h in *LysM-cre* ^*+*^ mice.

Neutrophil and alveolar macrophage Gal-3 levels were significantly reduced at both time points in *LysM-cre* ^*+*^ mice, whilst interstitial macrophage displayed reduced expression at 48 h. Expression was again found to be highest on alveolar macrophages and correlated with Gal-3 levels in BALf. Immunohistochemistry confirmed macrophage and neutrophil Gal-3 depletion (Supplementary Figure S3A). Reduced staining of Gal-3 in epithelial cells was also apparent in *LysM-cre* ^*+*^ mice.

We showed no significant differences in circulating neutrophil numbers following LPS administration in *LysM-cre* ^*+*^ and *Gal-3* ^*−/−*^ mice (Supplementary Figure S4) and no difference in neutrophil precursors in the bone marrow (Figure 1). In the BAL, although neutrophils were the predominant cell type following LPS in all genotypes there was a reduced overall number of neutrophils only in *Gal-3* ^*−/−*^ mice with no difference between *LysM-cre* ^*-*^ and *LysM-cre* ^*+*^ (Figure 3C). This suggests differences in the role of Gal-3 in the regulation of neutrophil extravasation and alveolar infiltration. Total neutrophil numbers in BAL following acute bleomycin were not determined.

### Mesenchymal Deletion of Gal-3 has No Effect on Bleomycin-Induced Fibrosis

**FIGURE 4:**
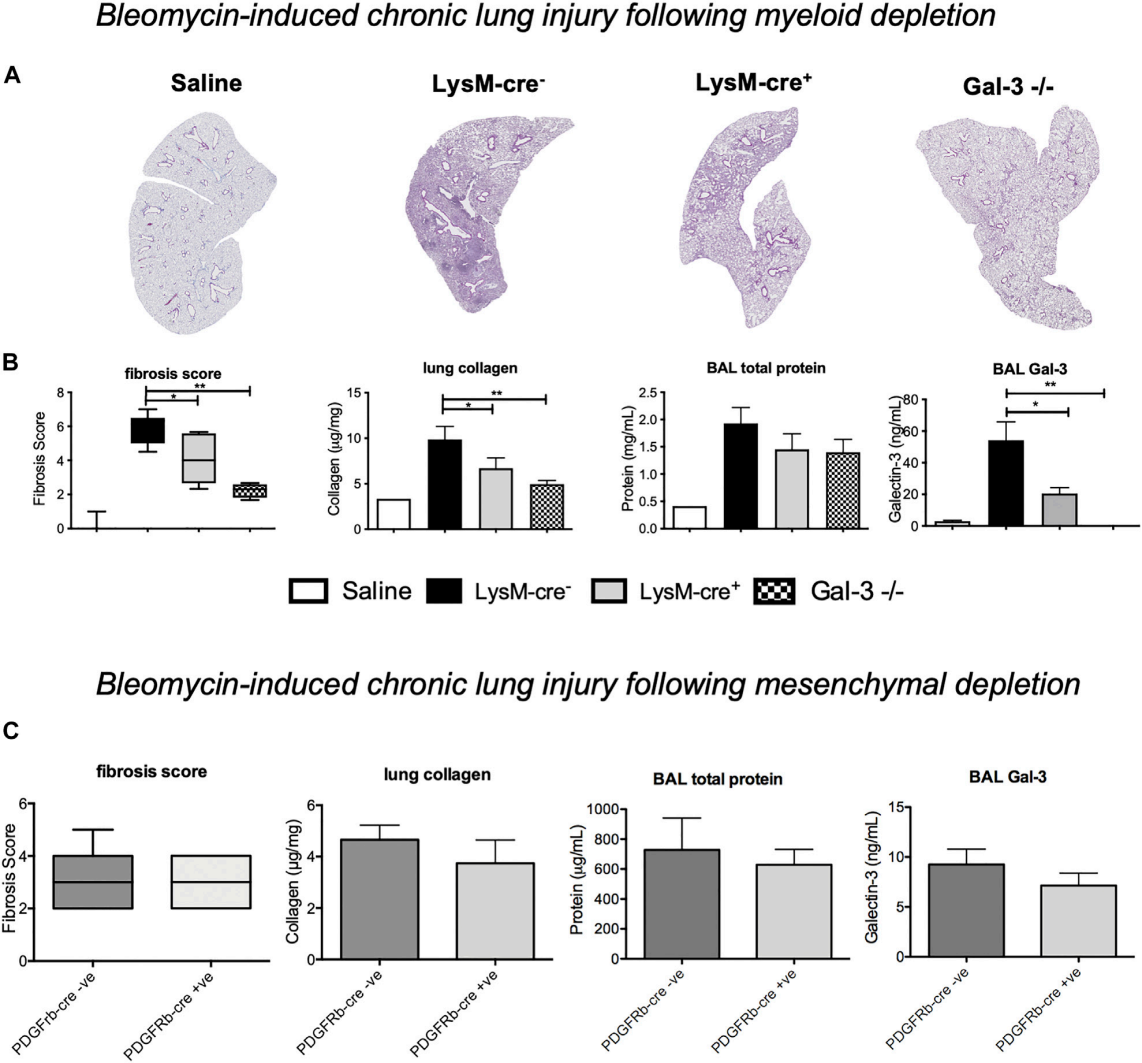
*- Effect of myeloid cell and myofibroblast Gal-3 depletion on bleomycin induced fibrosis*. Bleomycin was administered *i. t.* to *LysM-cre* ^*-*^ *, LysM-cre* ^*+*^ *, Pdgfrb-cre* ^*--*^ *Pdgfrb-cre* ^*+*^ mice or global *Gal-3* ^*−/−*^ mice. Lungs were retrieved on day 21. **(A)** Lung sections from *LysM-cre* ^*+/-*^ and *Gal-3* ^*−/−*^ mice 21 days after bleomycin administration, stained with Masson’s trichrome (purple) to identify fibrosis. **(B)** Fibrosis score, lung collagen, BAL total protein and BAL Gal-3 from *LysM-cre* ^*+/-*^ and *Gal-3* ^*−/−*^ mice 21 days after bleomycin administration. **(C)** Effect of mesenchymal cell Gal-3 depletion 21 days after bleomycin administration. Data represented as mean ± SEM. Analysed via 1-way ANOVA **(B)** or student’s *t*-test **(C)** (*n* = 4-5, **p < 0.01, ***p < 0.001).

Chronic bleomycin injury was then examined in *Pdgfrb-cre* mice to assess the contribution of mesenchymal cell (in particular, myofibroblast) Gal-3 on the development of pulmonary fibrosis. Immunofluorescence staining in *Pdgfrb-cre* ^*-*^ mice showed some co-expression of PDGFRβ and Gal-3 in mesenchymal cells however the majority of the Gal-3 positive cells were macrophages and epithelial cells and there was no co-localisation of PDGFRβ and Gal-3 in *Pdgfrb-cre* ^*+*^ mice (Supplementary Figure S2). No differences in fibrosis, BAL protein or Gal-3 levels were observed at 21 post-bleomycin administration following mesenchymal Gal-3 deletion (Figure 4C) suggesting myofibroblast Gal-3 is not a major contributor to fibrosis in the bleomycin model.

### Myeloid Cells Are a Key Contributor of Gal-3 That Drive Fibrosis

To determine if myeloid cell derived Gal-3 drives fibrosis, mice were treated with 33 μg bleomycin *i. t.* and examined at day 21 where peak fibrosis occurs. Histological analysis demonstrated a significant increase in pulmonary fibrosis following bleomycin administration, which was significantly reduced in *LysM-cre* ^*+*^ and *Gal-3* ^*−/−*^ mice (Figures 4A,B). Total lung collagen content was reduced from 9.41 ± 1.22 μg to 7.04 ± 0.90 μg collagen/mg of lung tissue in *LysM-cre* ^*+*^ compared to and *LysM-cre* ^*-*^ mice, and to 4.95 ± 0.42 μg collagen/mg of lung tissue in *Gal-3* ^*−/−*^ (Figure 4B). Levels of BAL total protein and BAL Gal-3 correlated with the levels of fibrosis. Myeloid deletion reduced Gal-3 levels in BAL by 60%.

## Discussion

Using transgenic mice with selective depletion of Gal-3 in myeloid or mesenchymal cell compartments, we assessed both the role and cellular source of Gal-3 in acute and chronic lung injury. Specific deletion of Gal-3 in *LysM-cre* ^*+*^ mice resulted in a decrease in Gal-3 expression primarily in CD11b^+^/CD24^+^/Ly-6G^+^ neutrophils and alveolar macrophages with only a partial reduction in expression on CD11b^+^/Ly-6C^+^ monocytes compared to mice with a global deletion in Gal-3. This resulted in a reduction in ALI and interstitial neutrophil recruitment. Previous work shows the *LysM-cre* driver targets alveolar macrophages and neutrophils with higher efficiency than infiltrating macrophages and monocytes (McCubbrey et al., 2017). In our studies alveolar macrophages displayed the highest positivity for Gal-3 and the reduction in BAL Gal-3 in *LysM-cre* ^*+*^ mice revealed alveolar macrophages as a key cellular source of Gal-3 in the inflamed lung. We hypothesise that alveolar macrophage derived Gal-3 plays a key role in neutrophil recruitment and found a reduction in the neutrophil chemoattractant G-CSF (Parsons et al., 2005) and IL-6, a typical biomarker of ALI (Castellani et al., 2019). A reduction in alveolar macrophage number was also observed following LPS. LPS has been shown to induce alveolar macrophage necrosis resulting in IL-1α release, endothelial cell activation and increased vascular permeability that allows neutrophils to infiltrate the lungs (Dagvadorj et al., 2015).

The fact that a partial reduction in Gal-3 was detected in BALf indicates that another cell type is contributing to Gal-3 levels in the alveolar space. Epithelial cells express Gal-3 (Pilette et al., 2007) yet are also affected by the *LysM-cre* driver, with recombination observed in a quarter of lung epithelial cells (McCubbrey et al., 2017). In our study, Gal-3 immunohistochemistry in the *LysM-cre* ^*+*^ mice show decreased staining in both recruited neutrophils and alveolar macrophages alongside a partial reduction in the epithelium. Flow cytometry analysis of lung digests confirmed a 35% reduction in Gal-3 levels in CD45^−^ cells following acute bleomycin.

Myeloid Gal-3 deletion reduced interstitial neutrophil recruitment yet had no significant impact on the % of circulating neutrophils nor the number of neutrophils recruited into the alveolar space when compared to *LysMcre* ^*-*^ mice. A reduction in total BAL neutrophils was only observed in Gal-3^−/−^ mice. This leads us to conclude that the lack of Gal-3 from myeloid cells (the key source of pulmonary Gal-3) mainly impacts extravasation of neutrophils from the circulation. This should be confirmed in bleomycin-induced ALI. Migration of neutrophils into the airways, although partially dependent on Gal-3, is not significantly altered in the *LysMcre* ^*+*^ mice, suggesting Gal-3 in the alveolar space derived from other cells (most likely AECs) is sufficient to recruit airway neutrophils. The impact of myeloid Gal-3 deletion on neutrophil extravasation from the vasculature vs migration into the alveolar space could be explained by differential effects on endothelial vs epithelial adhesion and migration. The ability of Gal-3 to facilitate adhesion is complex and dependent both on the local concentration of Gal-3 and the degree of cross linking and the repertoire of integrins expressed on endothelial and epithelial cells. In addition, Gal-3 can weaken cell–matrix adhesion by steric hindrance when it is bound either to integrins or their extracellular matrix ligands (Hughes, 2001). For endothelial migration, tight adhesion and cross-linking of neutrophils to the endothelium has been shown to be dependent on Gal-3 oligomerization (Sato et al., 2002). For epithelial migration, Gal-3 enables neutrophils to bind to laminin and migrate through the basement membrane into sites of inflammation (Kuwabara and Liu, 1996) and is required for optimal migration out of the vasculature and into the airways following *A. fumigatus* infection (Snarr et al., 2020). In this model, Gal-3 deficient mice were found to contain reduced BAL neutrophils compared to WT mice and, through the use of adoptive transfer studies, it was determined that Gal-3 from lung cells rather than neutrophil Gal-3 was necessary for effective neutrophil airway migration. This could in part explain why alveolar neutrophil recruitment was relatively unaffected in *LysMcre* ^*+*^ mice compared *LysMcre* ^*-*^ in the present study as epithelial Gal-3 could be driving recruitment of neutrophils into the alveoli. The precise mechanisms whereby Gal-3 regulates endothelial vs epithelial migration in the context of sterile ALI requires further study.

Lung conditions such as ALI and acute respiratory distress syndrome (ARDS) are driven by neutrophil-mediated lung injury and a reduction in pulmonary neutrophil number (e.g., *via* reduced recruitment or accelerated apoptosis) has been shown to be protective (Dorward et al., 2016, 2017; Humphries et al., 2018). Although non-activated neutrophils express relatively low levels of Gal-3, addition of exogenous Gal-3 induces neutrophil activation (Yamaoka et al., 1995; Kuwabara and Liu, 1996) and inhibits neutrophil apoptosis (Farnworth et al., 2008). Neutrophil activation has also been shown to be enhanced *in vitro* following LPS-induced Gal-3 oligomerization (Fermino et al., 2011), suggesting its inhibition is beneficial for reducing pro-inflammatory neutrophil activity following LPS-induced ALI. However, other reports have suggested Gal-3 acts as a negative regulator of LPS induced inflammation (Li et al., 2008; Nieminen et al., 2008). In this study a peri-lethal dose of LPS was administered by the intra-peritoneal route and is not a comparable ALI model.

Gal-3 also acts as an adhesion molecule following Streptococcal pneumonia infection to promote neutrophil recruitment (Sato et al., 2002; Nieminen et al., 2008) and elevated levels found in exudates have been shown to correlate with increased migration to inflammatory sites (Sano et al., 2000). Following *aspergillus* infection, extracellular Gal-3 enhances neutrophil motility and extravasation into the airways to reduce fungal burden (Snarr et al., 2020). While there is some evidence that Gal-3 may contribute to pathogen clearance in infection models, in sterile ALI augmented numbers of activated neutrophils and delayed rates of apoptosis would result in an overall exacerbation of tissue injury and failure of resolution.

Gal-3 expression is upregulated in activated myofibroblasts during hepatic fibrosis (Henderson et al., 2006). We therefore used the *Pdgfrb-cre* system, known to target lung myofibroblasts (Henderson et al., 2013), to delete Gal-3 expression in myofibroblasts. Reduced fibrosis in response to chronic bleomycin was observed following myeloid Gal-3 deletion whereas deletion within the mesenchymal compartment had little effect despite effective reduction in Gal-3 in PDGFRβ positive cells in *Pdgfrb-cre* ^*+*^ mice. This suggests that myeloid Gal-3 is a key driver of inflammation-induced fibrosis in the lung. Interestingly, therapeutic administration of the specific Gal-3 inhibitor GB0139 during the fibrotic phase of bleomycin injury was sufficient to reduce fibrosis after initial inflammation had resolved (MacKinnon et al., 2012). Inhibition of Gal-3 in AECs *in vitro* (either isolated from Gal-3^−/−^ mice or treated with GB0139) and in human AECs transfected with siRNA to Gal-3, resulted in a reduction TGFβ-induced beta-catenin activation and epithelial to mesenchymal transition (EMT) (MacKinnon et al., 2012). EMT has been suggested to be a mechanism that contributes to IPF pathogenesis, generating extra-cellular matrix accumulation contributing to scarring, although lineage tracing experiments have been unequivocal. Nevertheless, the epithelium appears to be a major producer of Gal-3 in the injured lung and may play an important role in acute, leading to chronic, inflammation. Inhibition of Gal-3 expression in these cells using an epithelial-driven *cre* system may help to address this question.

One limitation of this study is that only surface Gal-3 was measured so it is conceivable that intracellular Gal-3 could be contributing to some of the effects seen here. The use of inhibitors that function exclusively extracellularly *via* binding to the Gal-3 CRD domain vs cell permeable inhibitors acting in a glycan independent fashion will help will answer this question. These studies are underway. Studies with the predominately extracellular Gal-3 inhibitor GB0139 delivered by the *i. t.* route have shown effective reduction in bleomycin-induced fibrosis (MacKinnon et al., 2012). Results from a Phase I clinical trial in IPF patients with GB0139 demonstrate a way to target the lung directly to reduce Gal-3 expression (Hirani et al., 2021).

Gal-3 inhibition may offer protection both in the acute phase and in the longer-term fibrotic sequelae following injury. This work identifies alveolar macrophages as a significant source of Gal-3 that drives inflammation and the development of pulmonary fibrosis and confirms direct targeting of Gal-3 in the lung as an attractive novel therapy for both acute lung injury and chronic lung disease.

## Data Availability

The raw data supporting the conclusions of this article will be made available by the authors, without undue reservation.

## Supplementary Material

Supplementary Figure S1 Gal-3 expression assessment in other pulmonary leukocyte subsets. Mice received 33 µg bleomycin and were retrieved at 24 h **(A)** Frequency of Ly-6C^low^ monocytes, eosinophils, CD103^+^ DCs and CD11b^+^ DCs following bleomycin. **(B)** Gal-3 expression. **(C)** Gating strategy to identify B cells (CD45^+^ CD3^−^ B220^+^), CD4^+^ T cells (CD45^+^ CD3^+^ CD4^+^) or CD8^+^ T cells (CD45^+^ CD3^+^ CD8^+^) in pulmonary interstitium. d) Gal-3 expression in CD45^−^ cells. Data represented as mean ± SEM. Analysed via 1-way ANOVA (*n* = 4–5, ***p* < 0.01, ****p* < 0.001).

Supplementary Figure S2 *G-CSF, IL-6, IL-10 in BALf from LysM-cre*^*-*^, *LysM-cre*^*+*^ and *Gal-3* ^*−/−*^ *mice following bleomycin-induced lung injury (24 h).* Data represented as mean ± SEM. Analysed *via* 1-way ANOVA (*n* = 4-5, ***p* < 0.01, ****p* < 0.001).

Supplementary Figure S3 *Gal-3 immunohistochemistry/immunofluorescence in LysM-cre and Pdgfrb-cre mice.****(A)****LysM-cre Gal-3 conditional knockdown in LPS-induced lung injury (24 h)*. b) PDGFRβ and Gal-3 co-staining in *Pdgfrb-cre mice following bleomycin-induced lung injury (21* *days)*. (Gal-3, green; PDGFRβ, red; DAPI, blue).

Supplementary Figure S4 *Profile of neutrophils in blood and BAL neutrophil Gal-3 expression in LysM-cre*^*-*^*, LysM-cre*^*+*^ and *Gal-3* ^*−/−*^ mice *following LPS-induced lung injury (24 h).* Data represented as mean ± SEM. Analysed via 1-way ANOVA (*n* = 4-5, ***p* < 0.01, ****p* < 0.001).
