# Supplementary figures and images for: Selective Myeloid Depletion of Galectin-3 Offers Protection Against Acute and Chronic Lung Injury
Source: Front Pharmacol. 2021 Aug 30;12:715986. doi: 10.3389/fphar.2021.715986 (PMC8435800; doi:10.3389/fphar.2021.715986)

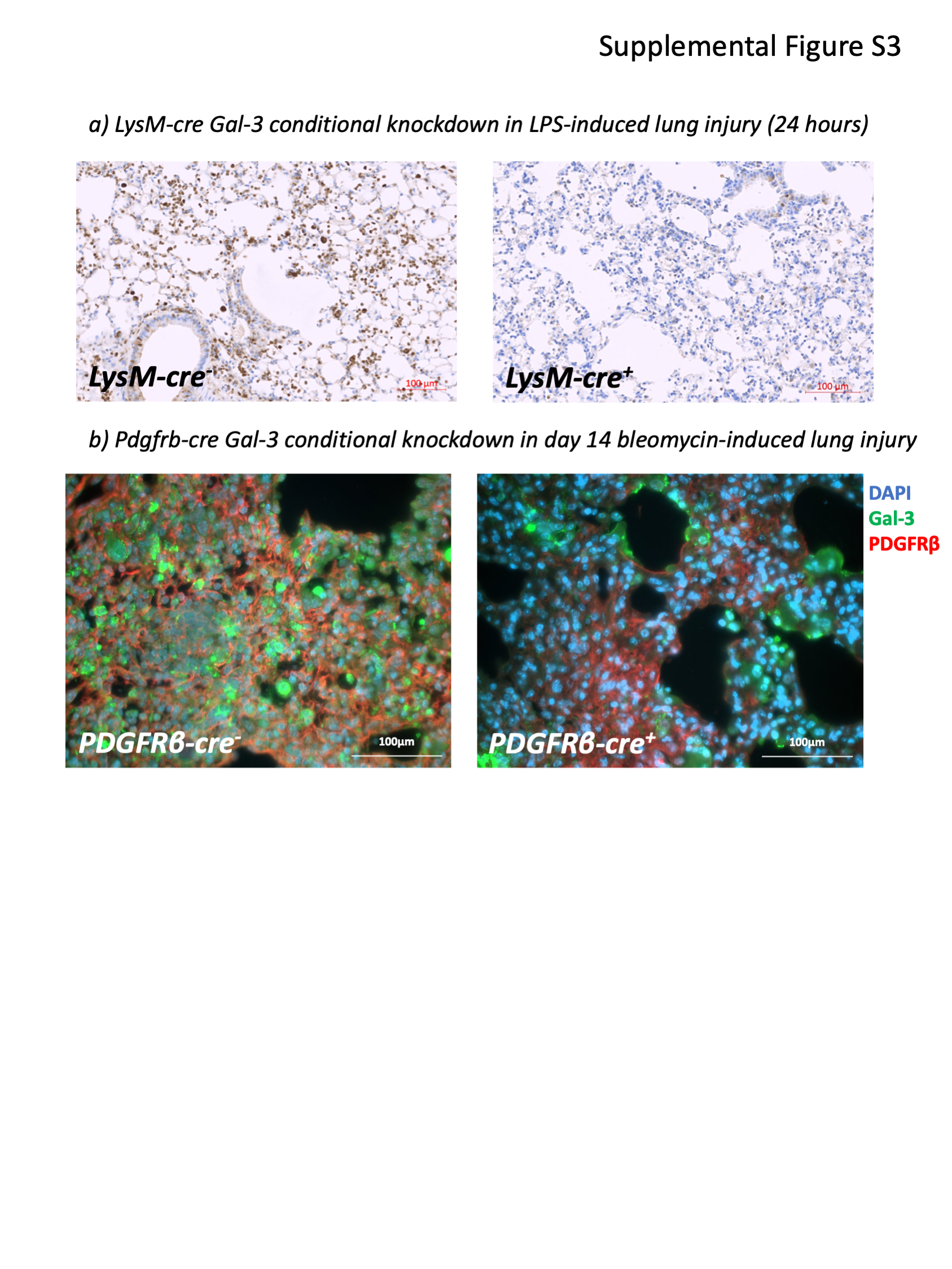

Supplement: Supplementary file 1 [file Image3.TIFF]

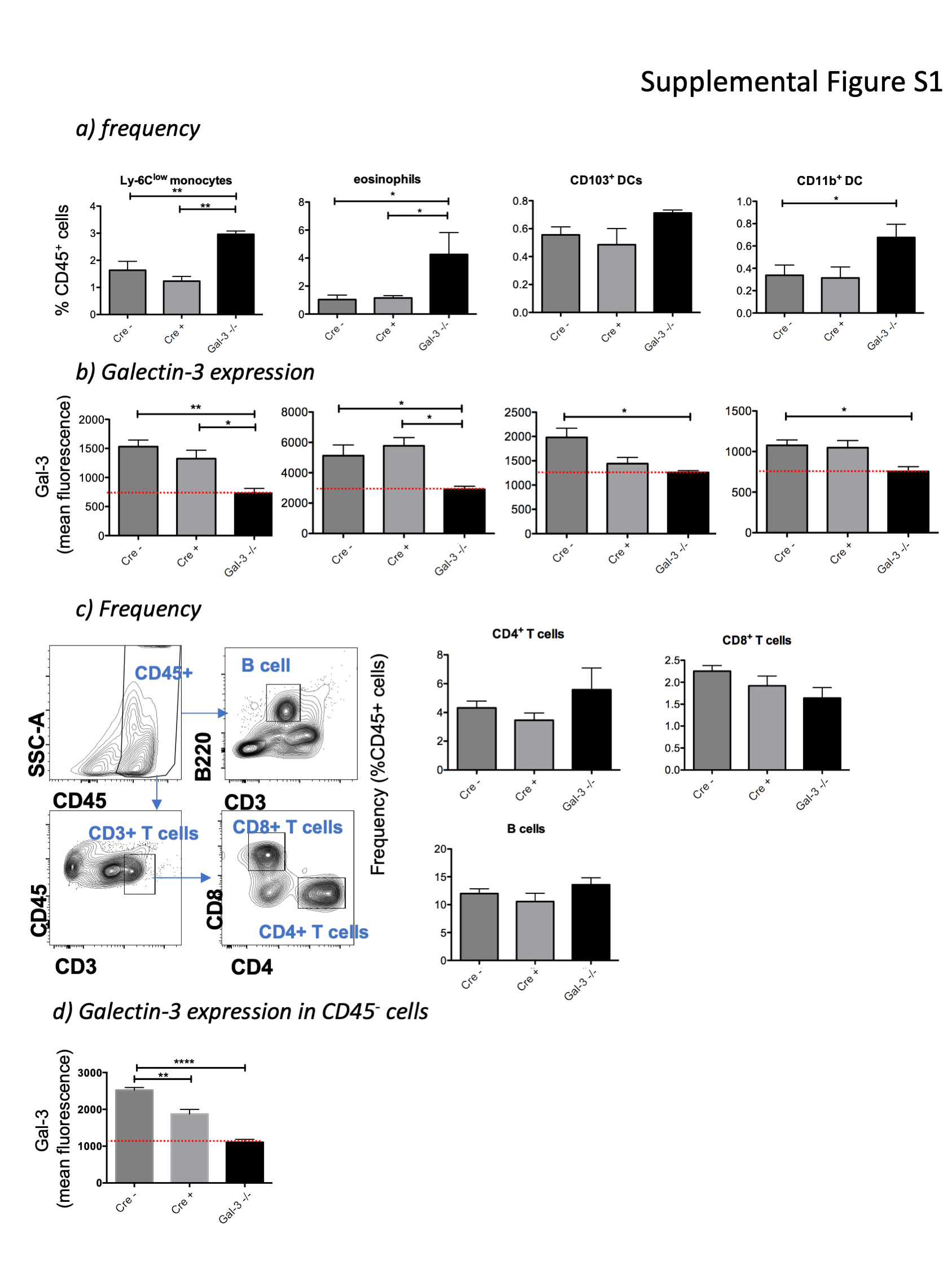

Supplement: Supplementary file 2 [file Image1.TIFF]

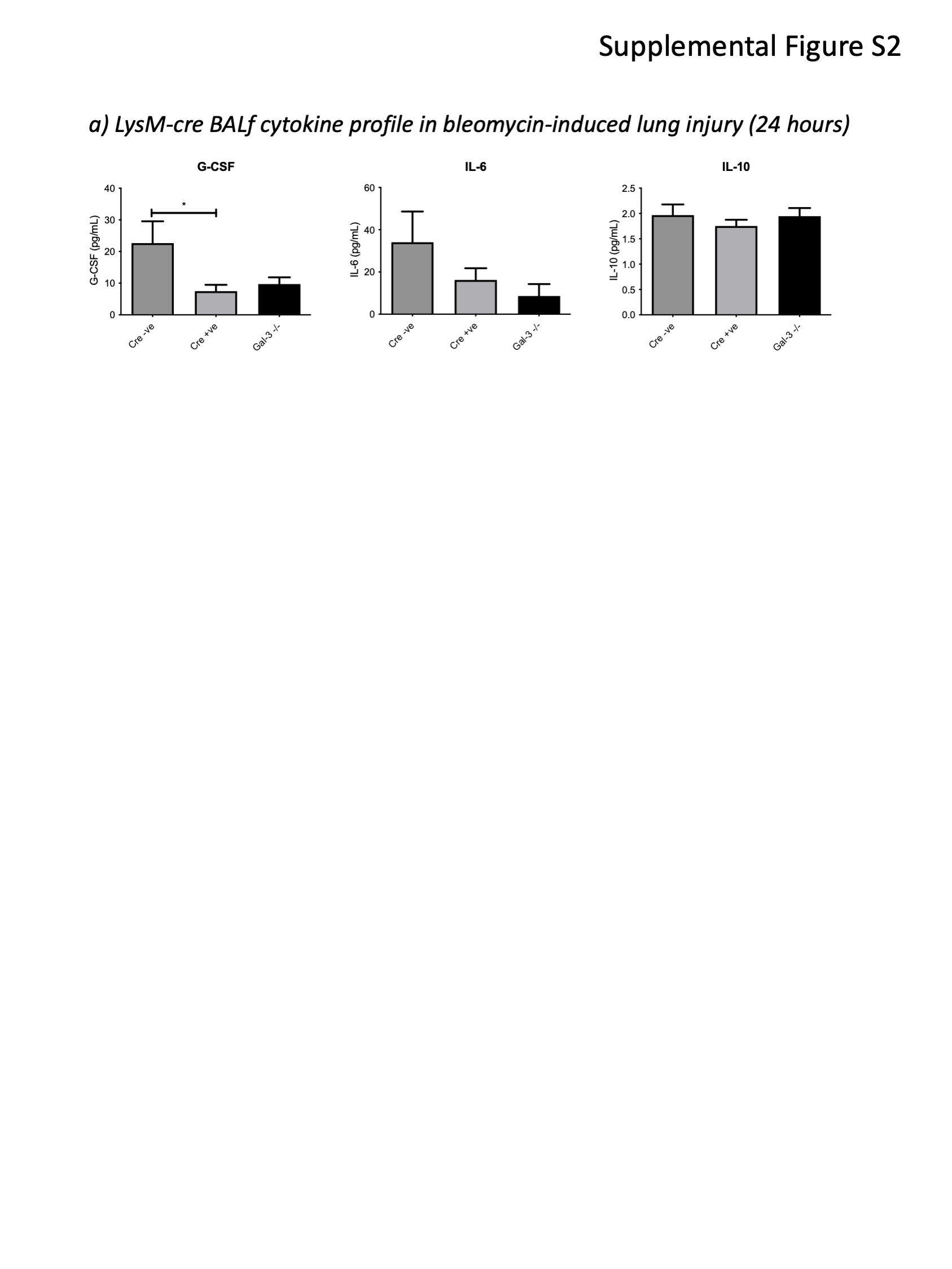

Supplement: Supplementary file 3 [file Image2.TIFF]

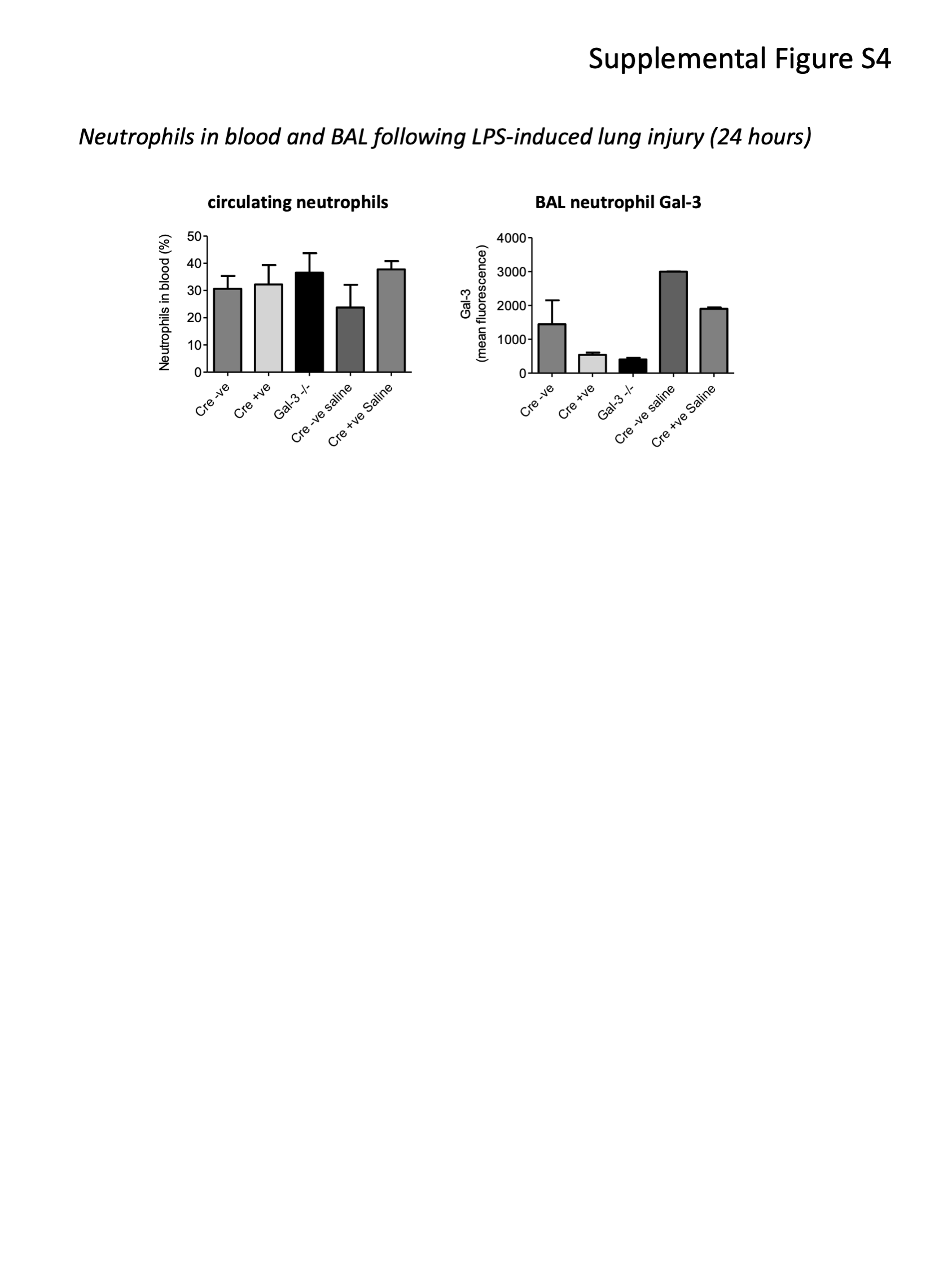

Supplement: Supplementary file 4 [file Image4.TIFF]
